# Expression Analysis of Genes Involved in the RB/E2F Pathway in Astrocytic Tumors

**DOI:** 10.1371/journal.pone.0137259

**Published:** 2015-08-28

**Authors:** Wallax Augusto Silva Ferreira, Mariana Diniz Araújo, Nilson Praia Anselmo, Edivaldo Herculano Correa de Oliveira, José Reginaldo Nascimento Brito, Rommel Rodriguez Burbano, Maria Lúcia Harada, Bárbara do Nascimento Borges

**Affiliations:** 1 Francisco Mauro Salzano Molecular Biology Laboratory, Institute of Biological Sciences, Federal University of Pará (Universidade Federal do Pará—UFPA)–Belém, Pará, Brazil; 2 Tissue Culture and Cytogenetics Laboratory, Evandro Chagas Institute (Instituto Evandro Chagas)–Belém, Pará, Brazil; 3 Ophir Loyola Hospital–Belém, Pará, Brazil; 4 Human Cytogenetics Laboratory, Institute of Biological Sciences, UFPA–Belém, Pará, Brazil; 5 Agricultural Technology Center, Socio-environmental and Water Management Institute, Federal Rural University of Amazônia (Universidade Federal Rural da Amazônia)–Belém, Pará, Brazil; University of Navarra, SPAIN

## Abstract

Astrocytic gliomas, which are derived from glial cells, are considered the most common primary neoplasias of the central nervous system (CNS) and are histologically classified as low grade (I and II) or high grade (III and IV). Recent studies have shown that astrocytoma formation is the result of the deregulation of several pathways, including the RB/E2F pathway, which is commonly deregulated in various human cancers via genetic or epigenetic mechanisms. On the basis of the assumption that the study of the mechanisms controlling the INK4/ARF locus can help elucidate the molecular pathogenesis of astrocytic tumors, identify diagnostic and prognostic markers, and help select appropriate clinical treatments, the present study aimed to evaluate and compare methylation patterns using bisulfite sequencing PCR and evaluate the gene expression profile using real-time PCR in the genes *CDKN2A*, *CDKN2B*, *CDC6*, *Bmi-1*, *CCND1*, and *RB1* in astrocytic tumors. Our results indicate that all the evaluated genes are not methylated independent of the tumor grade. However, the real-time PCR results indicate that these genes undergo progressive deregulation as a function of the tumor grade. In addition, the genes *CDKN2A*, *CDKN2B*, and *RB1* were underexpressed, whereas *CDC6*, *Bmi-1*, and *CCND1* were overexpressed; the increase in gene expression was significantly associated with decreased patient survival. Therefore, we propose that the evaluation of the expression levels of the genes involved in the RB/E2F pathway can be used in the monitoring of patients with astrocytomas in clinical practice and for the prognostic indication of disease progression.

## Introduction

Astrocytomas are tumors derived from glial cells known as astrocytes. They are considered the most common primary neoplasias of the central nervous system (CNS) and differ in their location, age, size, invasive potential, morphology, tendency to progress, and clinical course [1]. According to the World Health Organization (WHO), astrocytomas can be classified into four grades: pilocytic astrocytoma (grade I), low-grade astrocytoma (grade II), anaplastic astrocytoma (grade III) and glioblastoma multiforme (GBM) (grade IV), the latter being the most aggressive and malignant form [1–3].

Despite the advancements in diagnostic and therapeutic strategies, astrocytic tumors remain a challenge for medicine. Current treatments for such tumors include radiotherapy, chemotherapy, and surgical resection. However, the response to these treatments is still poor, and the median survival of patients with the most aggressive tumor type rarely reaches two years [4, 5].

As with most cancer types, astrocytomas develop because of genetic and epigenetic changes that accumulate as the tumor progresses [1, 6, 7]. However, limited data are available on the molecular changes that occur in most astrocytoma grades, of which GBM is the most studied. Previous genomic studies have indicated that the formation of GBMs results from the deregulation of three main pathways: the phosphatidylinositol 3-kinase (PI3K)/tyrosine kinase (RTK) PI3K/AKT pathway, p53, and RB/E2F [6, 8, 9].

The RB/E2F pathway coordinates several important biological processes, including cell migration and differentiation, development, apoptosis, mitosis, DNA replication and repair, and cell cycle checkpoints [10, 11,12]. This pathway is composed of five protein families: INK4 family (p16^INK4A^, encoded by the *CDKN2A* gene; p15^INK4B^, encoded by the *CDKN2B* gene; p18^INK4C^; and p19^INK4D^), D-type cyclins (cyclins D1, D2, and D3), cyclin-dependent protein kinases (CDK4 and CDK6), RB family proteins (RB, p107, and p130), and transcription factors of the E2F family (heterodimers of E2F1-8 with DP1-2) [13].

Polycomb Group (PcG) proteins form large multimeric complexes which are involved in gene silencing by chromatin organization changes [14]. They can be divided into two major groups: Repressive Complex Policomb 1 (PRC1) and Repressive Complex Policomb 2 (PRC2) [15]. The *Bmi-1* gene is a member of the Polycomb 1 (PcG1) gene cluster and functions as a transcriptional repressor of several genes via acetylation, methylation, and mono-ubiquitination of histones and methylation of chromatin [14]. Furthermore, some studies show that the *Bmi-1* gene is also related in self-renewal and differentiation of normal and tumor stem cells [16, 17], prevents senescence and immortalizes cells by activating telomerase [18], hematopoiesis [19], neural and skeletal development [19], cell cycle [20], and protection against oxidative stress and DNA damage [21].

One of the most studied pathways of *Bmi-1* that is associated with cancer is the RB/E2F pathway. The *Bmi-1* directly or indirectly represses the transcription of *CDKN2A* and/or *p14* ^*ARF*^ in a dose-dependent manner, and consequently promotes increased cell proliferation [22]. The promoter silencing and consequent loss of INK4A/ARF locus expression has been important for both the progression and prognosis of various hematological cancers [23, 24].

The human *CDC6* gene codes for an AAA^+^ ATPase that binds to the replication origin recognition complex (ORC) and facilitates the recruitment of the mini-chromosome maintenance (MCM) complex [25]. High levels of *CDC6* can transcriptionally inactivate the INK4/ARF locus [26]. *CDC6* binds specifically to the regulatory domain (RD) of this locus, recruits histone deacetylases (HDACs), particularly HDAC1 and HDAC2, and induces the heterocromatinization, suppressing the entire locus. Additionally, it recruits *Bmi-1* to the regulatory domain (RD) of INK4/ARF locus repressing the entire locus [27].

Deregulation of components of the RB/E2F pathway via genetic and epigenetic changes occurs frequently in most cancer types, including gastrointestinal tract endocrine tumors [28], adenocarcinoma [29, 30], basal cell carcinoma [31], and astrocytomas [6, 32], making it an important target in oncology studies.

Therefore, the present study aimed to evaluate the expression and methylation profiles of the genes *CDKN2A*, *CDKN2B*, *CDC6*, *Bmi-1*, *CCND1*, and *RB1* in astrocytic tumors in the northern region of Brazil.

## Materials and Methods

### Collection of tumor samples and study approval by the Research Ethics Committee

This study was approved by the Research Ethics Committee of the Health Sciences Institute (Instituto de Ciências da Saúde–ICS) of UFPA (Process No. 025/06), and the use of nervous system samples was approved by the Research Ethics Committee of the Ophir Loyola Hospital, and a written informed consent was obtained from all patients.

Astrocytoma samples were obtained from biopsies of patients from the Ophir Loyola Hospital, Belém, Pará, Brazil. Additionally, ten non-neoplastic samples were collected in the same Hospital. All tissue samples were immediately frozen in liquid nitrogen and stored in RNAlater (Life Technologies) at -70°C until the extraction stage. Tissue samples were collected and stored by the research group of the Francisco Mauro Salzano Laboratory of Molecular Biology at the Federal University of Pará (Universidade Federal do Pará –UFPA).

### Sample processing

#### Extraction and quantification of genomic DNA and total RNA

To obtain total RNA, tissue samples were homogenized in liquid nitrogen and subjected to the chemical extraction of total RNA using TRIzol (Invitrogen Life Technologies) according to the specifications provided by the manufacturer. The quality of RNA samples was assessed by electrophoresis on 3% agarose gels stained with ethidium bromide. The RNA samples were quantitated using a NanoDrop ND-1000 spectrophotometer (Thermo Scientific, Rockford, IL, USA) and stored at -70°C.

Genomic DNA was extracted using a standard protocol with phenol-chloroform-isoamyl as previously described by Sambrook & Russell [33].

The DNA concentration was measured using a NanoDrop ND-1000 spectrophotometer (Thermo Scientific, Rockford, IL, USA), and the integrity of the extracted genomic DNA was assessed by electrophoresis on 1% agarose gels stained with ethidium bromide.

#### cDNA synthesis and real-time PCR

For the cDNA synthesis, we used the High-Capacity cDNA Reverse Transcription kit (Applied Biosystems), following the manufacturer's instructions.

The expression of the genes *CDKN2B*, *CDKN2A*, *CDC6*, *Bmi-1*, *CCND1*, and *RB1* was quantitated by real-time PCR using the Taqman system (Applied Biosystems, Foster City, CA, USA). The cDNA samples were amplified using an ABI 7500 Fast thermocycler (Applied Biosystems, Foster City, CA, USA) and Taqman Gene Expression Assays (Applied Biosystems, Foster City, CA, USA).

All reactions were performed in triplicate. For the analysis of real-time PCR data, the relative gene expression was calculated using cycle threshold (C_t_) values, which were converted into relative expression values according to the 2^-ΔΔCT^ method. The C_t_ values of GAPDH transcripts (endogenous control of gene expression in non-neoplastic astrocytes of the human temporal lobe) were used for normalization.

#### Treatment of DNA with sodium bisulfite and bisulfite sequencing PCR (BSP)

To evaluate the correlation between the gene expression levels obtained by real-time PCR and the methylation pattern, a fragment of the promoter region of the genes studied was sequenced using BSP. For this purpose, the genomic DNA was chemically modified using sodium bisulfite according to the protocol described by Herman et al. [34]. This treatment promotes the deamination of non-methylated cytosines, converting them to uracil, whereas methylated cytosines are not affected. Subsequently, the samples were purified using the Wizard DNA Purification kit (Promega), following the manufacturer's instructions, and the DNA samples were resuspended in 50 μL of ultrapure water and stored at -20°C until use.

For the primer design, we analyzed the sequences of the promoter regions 1.0 kb upstream and 0.5 kb downstream from the transcription initiation sites of each gene using the software Methyl Primer Express v1.0 (Applied Biosystems). Each PCR was performed in a final reaction volume of 25 μL containing 2 μL of sodium bisulfite-treated DNA; 2.5 μL of 10X reaction buffer; 1 μL of MgCl_2_ (1.5 mM); 0.5 μL of each dNTP (10 mM) (Life Technologies); 1 μL of each primer (10 mM); 0.3 μL of Taq DNA polymerase (Promega, Inc.); and ultrapure H_2_O to complete the final reaction volume.

The amplification conditions and the primers used for each gene are detailed in Table 1.

**Table 1:** Primers used in this study, with their respective sequences, annealing temperatures, amplicon size and references.

Gene	Primer sequences (5’–3’)	Annealing temperature (°C)	Amplicon size	Source
***CDC6***	F 5’AAGATTTGGGGTTTTTTTATTG 3’	57	270 pb	This study
	R 5’CCTCAATACAAAATCCTTCTCA 3’			
***RB1***	F 5’TTTTAAGTTTGTTTTTGTTTTGG 3’	52	280 pb	This study
	R 5’TCATAAAAAATTAAACTAAAAAACCT 3’			
***Bmi-1***	F 5’TGTAGAAAGGTTTTTAGATGTTGG 3’	57	377 pb	This study
	R 5’CCTCAAACAAAACTAAACACCTT 3’			
***CDKN2B***	F:5’GGTTGGTTTTTTATTTTGTTAGAG 3’	55	210 pb	Kawamata et al. [35]
	R: 5’CCTTCCTAAAAAACCTAAACTCAA 3’			
	Fext: 5’GGGGTTAGGGTTAGGTAGG 3’			
	Rext: 5'AACTACACCAATACAACCACATA 3’			
***CDKN2A***	F1: 5’GAGGGATAGGGTCGGAGGGGGTTT 3’	58	178 pb	Borges et al. [36]
	R2: 5’TACAAACCCTCTACCCACCT 3’			
	F2: 5’GAAGAAAGAGGAGGGGTTGGTTGG 3’			
	R1: 5’TAAACAACGCCCCCGCCTCCAACAA3’			

F: Forward, R: Reverse, Fext: External forward, Rext: External reverse.

The PCR amplification products were analyzed on 3% agarose gels stained with ethidium bromide and subsequently purified using the EZ-10 Spin Column PCR Product Purification kit (Bio Basic/Ludwig Biotec), following the manufacturer's instructions.

The purified DNA was sequenced using the dideoxy chain-termination method according to the methodology described by Sanger *et al*. [37]. The sequencing reaction was performed using the BigDye Terminator Cycle Sequencing Standard kit (Applied Biosystems) version 3.1 and analyzed on an ABI 3130 automated sequencer (Applied Biosystems).

The obtained sequences were aligned manually using the software BioEdit version 7.0.9 [38]. The methylation patterns of gene sequences were analyzed using the software BISMA (Bisulfite Sequence DNA Methylation Analysis) [39] and BiQ Analyzer [40]. In this study, we considered a gene hypermethylated when >15% of the CpG islands examined were methylated.

To verify the accuracy of the BSP sequencing, we included in each run of all genes the control DNA from the EpiTect PCR Control DNA Set (Qiagen) that had contained both bisulfite-converted 100% methylated as well as unmethylated DNA as positive controls and unconverted unmethylated DNA as a negative control.

### Statistical analysis

To analyze the BSP data, differences in the methylation frequencies among the tumor grades and the association between the methylation of the promoter region/mRNA and clinical parameters (age, gender, and tumor grade) were evaluated using the chi-squared test.

For the real-time PCR analysis, differences among the groups were analyzed using a non-parametric Kruskal-Wallis test followed by Dunn’s comparison. Fisher's exact test was used to evaluate the association between survival and the level of gene expression, and the samples were divided into two groups, low survival rate (<24 months) and high survival rate (≥24 months), following the method proposed by Skiriutè et al. [41]. The statistical significance of the tests was set at p<0.05.

The association of the expression levels of the studied genes was analyzed using a non-parametric Spearman’s rho test. A correlation coefficient (r) ≥ 0.7 indicated a strong correlation, r < 0.7 and r ≥ 0.3 indicated a moderate correlation, and r < 0.3 indicated a weak correlation. Positive r values indicated proportional magnitudes, whereas negative r values indicated inversely proportional magnitudes.

All statistical analyses were performed using GraphPad Prism 5 version 5.01 (GraphPad Software, Inc., USA), and the statistical significance of all tests was set at p<0.05.

## Results

### Clinical data

The 58 analyzed samples were from patients with a mean age of 47.06 years (range 14–83 years). Of the 58 samples, 22 cases were classified as grade II (37.9%), with a mean age of 36.09 years (14–64 years), 13 cases were classified as grade III (22.4%), with a mean age of 42.84 years (18–61 years), and 23 cases were designated grade IV (39.6%), with a mean age of 59.95 years (26–83 years). Of the 58 cases, 35 were men (60.03%), and 23 were women (39.97%).

### The relative expression of *CDKN2A*, *CDKN2B*, and *RB1* is decreased in astrocytomas

**Fig 1:**
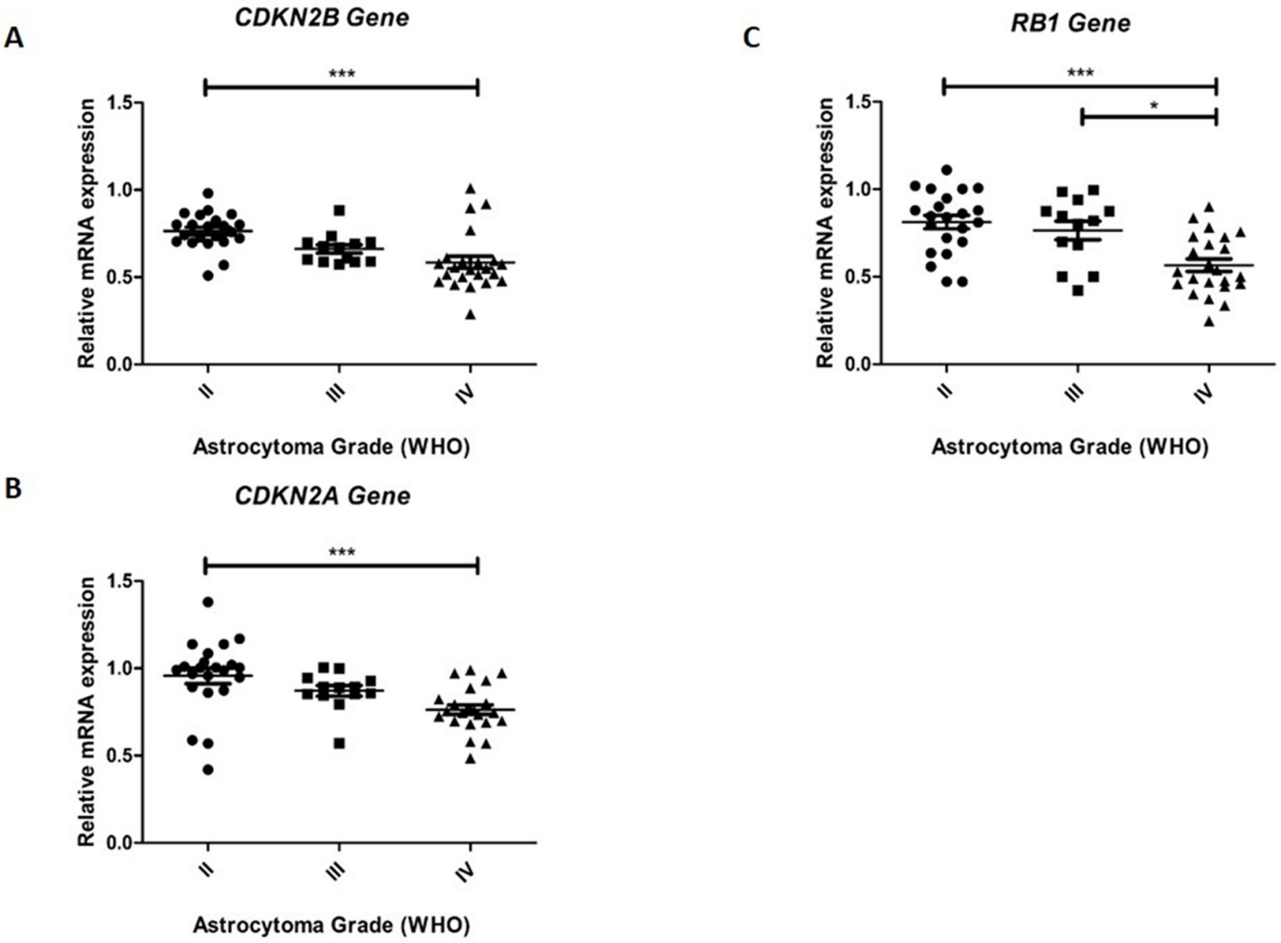
Relative expression levels of the genes *CDKN2B*, *CDKN2A*, and *RB1* in different astrocytoma grades. Real-time qRT-PCR was used to determine the relative *CDKN2B* (A), *CDKN2A* (B), and (C) *RB1* mRNA levels in astrocytomas. The expression of these genes reduces with increasing grade of malignancy. Data are expressed as fold-change in mRNA expression compared with that of a normal brain control (the relative expression of the control equals 1). The horizontal lines indicate the median expression level in each tumor grade. Statistical significance is denoted by * (p≤0.05), ** (p<0.01) and *** (p<0.001).

The relative expression of the genes *CDKN2A*, *CDKN2B*, and *RB1* was significantly decreased in all the examined tumor grades. Pairwise comparison between the tumor grades indicated that the relative expression of the *CDKN2A* and *CDKN2B* transcripts was approximately 1.3-fold lower in grade IV glioblastoma compared with that in grade II astrocytoma (p<0.001) (Fig 1A and 1B). The pairwise comparison indicated no significant differences in the relative expression of both genes between grade II and grade III glioblastomas.

The relative expression of the *RB1* transcript was 1.6-fold lower in glioblastoma samples compared with grade II samples and 1.4-fold lower compared with grade III samples (Fig 1C).

These results indicate that the underexpression of these genes is stage-specific and is more evident in high-grade tumors (WHO grade IV); consequently, underexpression of these genes may be associated with the aggressiveness of astrocytic tumors.

No significant differences were observed in the relative expression of the genes *CDKN2B*, *CDKN2A*, and *RB1* as a function of gender, age, and survival.

### The relative expression of *Bmi-1*, *CCND1*, and *CDC6* is increased in astrocytomas

The relative expression of the genes *Bmi-1*, *CCND1*, and *CDC6* was significantly increased in samples from all the tumor grades analyzed, and grade IV tumors were the most frequent.

**Fig 2:**
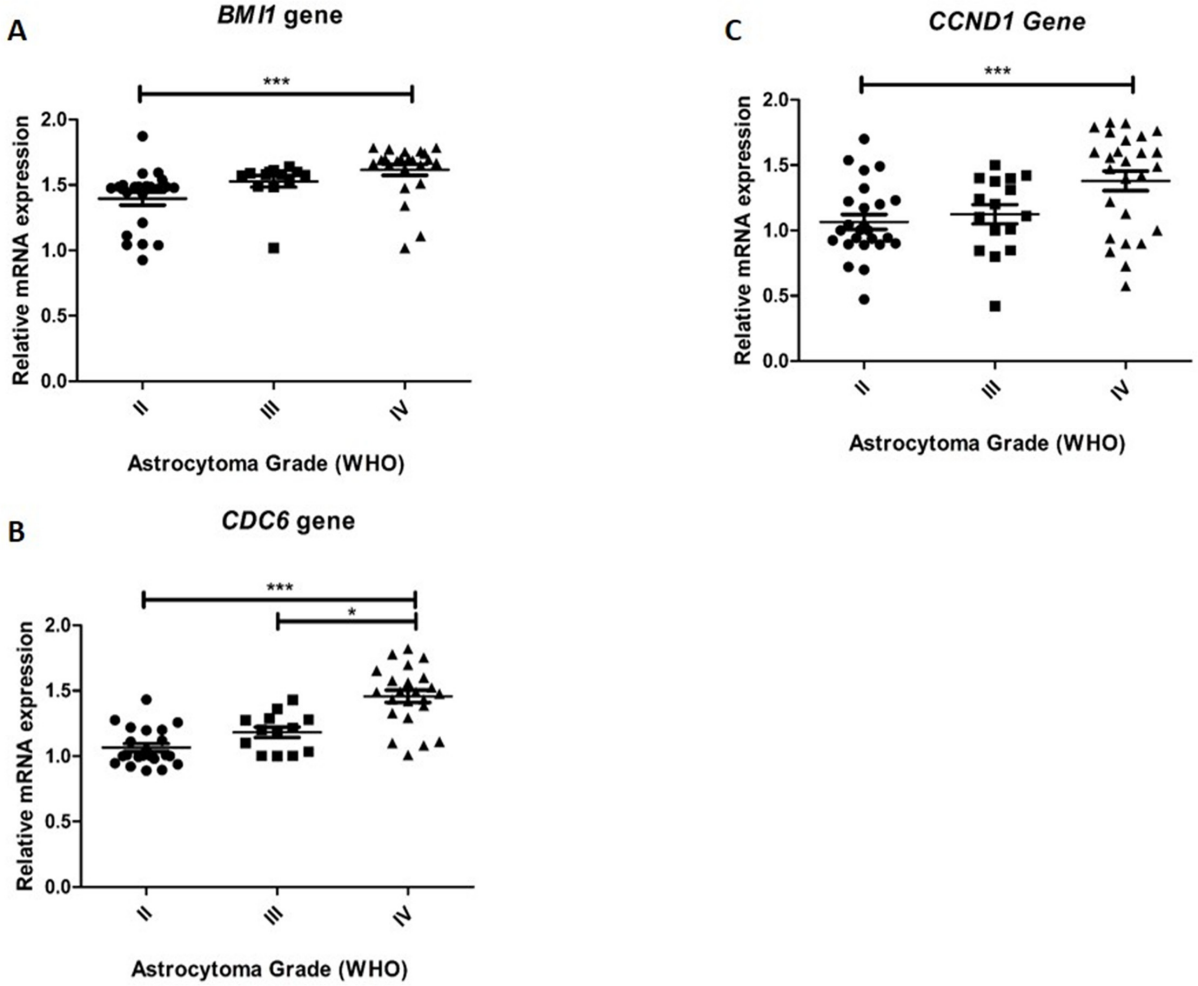
*Bmi-1*, *CDC6*, and *CCND1* relative expression analysis. Real-time qRT-PCR was used to determine the relative ***Bmi-1* (A), *CDC6* (B), and *CCND1* (C)** mRNA levels in astrocytomas. The expression of these genes was highly upregulated in GBM (Grade IV) as compared to grades II and III. Data are expressed as the fold-change in mRNA expression compared with that of a normal brain control (the relative expression of the control equals 1). The horizontal lines indicate the median expression level in each tumor grade. Statistical significance is denoted by * (p≤0.05), ** (p<0.01) and *** (p<0.001).

Significant differences were observed in the relative expression of *Bmi-1* (Fig 2A) and *CCND1* (Fig 2C) between grades II and IV astrocytomas (p<0.001 for both), with a 1.15-fold and 1.31-fold increase in their expression in GBM samples, respectively. In addition, significant differences were observed in the expression of *CDC6* between grade II and IV astrocytomas (p<0.0001), with a 1.38-fold increase in its relative expression in GBM samples, and between levels III and IV astrocytomas (p<0.05), with a 1.27-fold increase in its gene expression in GBM samples (Fig 2B). Although no significant differences were observed as a function of age and gender, a significant association was found between increased transcript expression and decreased patient survival (*Bmi-1*, p = 0.0002; *CDC6*, p<0.0001; *CCND1*, p<0.0001).

### Gene expression association in different astrocytoma grades

**Fig 3:**
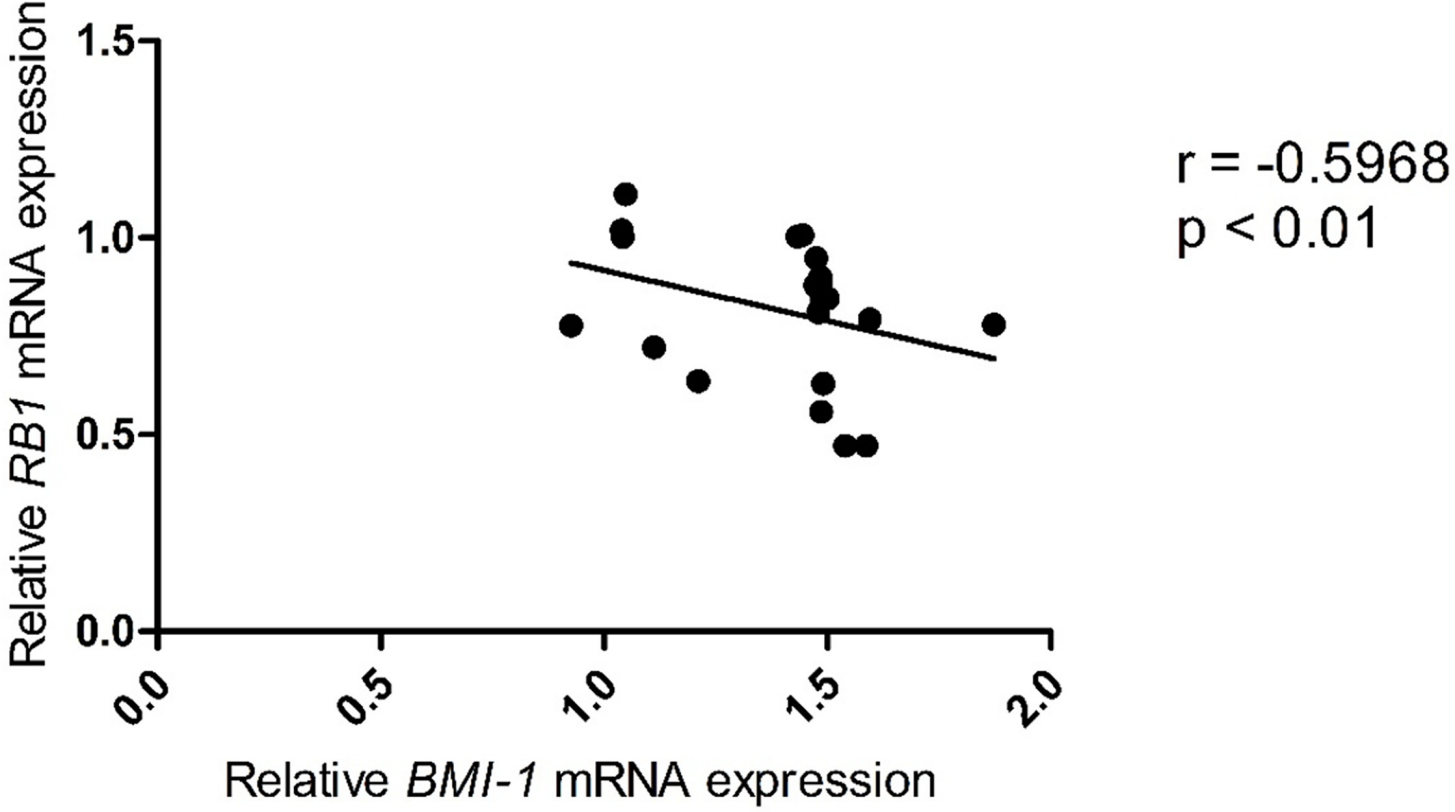
Correlation of the relative expression levels of *Bmi-1* and *RB1* in grade II astrocytomas. Analyzing the samples belonging to grade II astrocytomas, we found that the only significant negative correlation was between *Bmi-1* and *RB1* genes.

The relative expression of *CDKN2B*, *CDKN2A*, *CDC6*, *Bmi-1*, *CCND1* and *RB1* was analyzed using pairwise combinations (Table 2). In grade II astrocytomas, a moderate inverse correlation was observed only between the expression of *RB1* and *Bmi-1* (r = -0.5968, p = 0.001) (Table 2, Fig 3).

**Table 2:** Association of gene expression levels evaluated using pairwise combinations in grade II astrocytomas.

	*Bmi-1*	*CDC6*	*CDKN2B*	*CDKN2A*	*RB1*	*CCND1*
***Bmi-1***						
***CDC6***	0.1073					
***CDKN2B***	-0.3394	-0.1587				
***CDKN2A***	-0.1553	-0.2508	0.3326			
***RB1***	-0.5968**	0.05874	0.2908	0.2739		
***CCND1***	-0.002823	0.3186	0.2084	0.1383	0.2693	

Spearman's rho. (*p≤0.05, **p<0.01; ***p<0.001).

**Fig 4:**
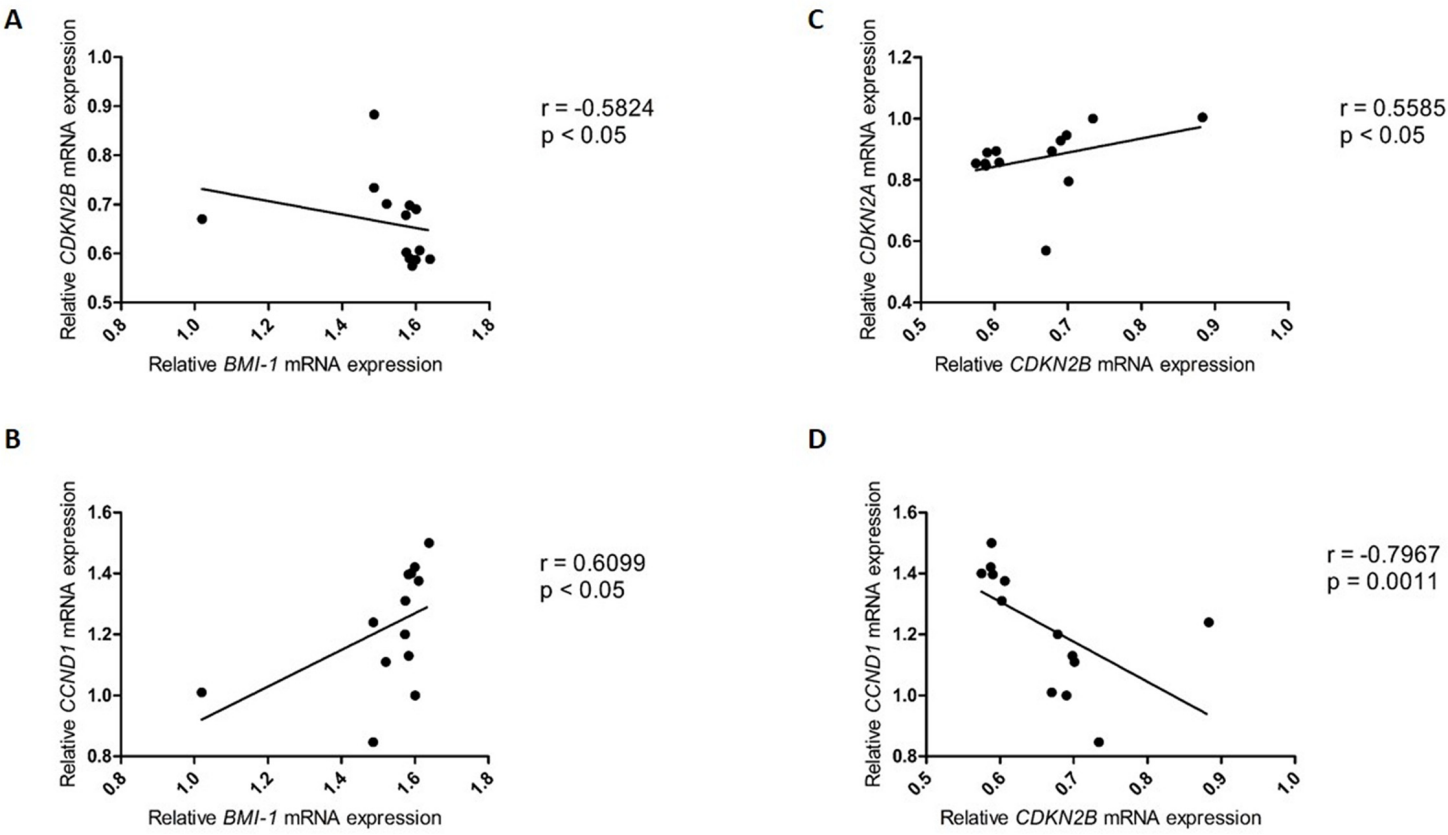
Correlation of the relative expression levels in grade III astrocytomas. **(A)** *Bmi-1* and *CDKN2B*; **(B)** *Bmi-1* and *CCND1* **; (C)** *CDKN2B* and *CDKN2A*; and **(D)** *CDKN2B* and *CCND1*.

In grade III astrocytomas, there was a moderate correlation between the relative expression levels of *Bmi-1* and *CDKN2B* (r = -0.5824, p<0.05) (Fig 4A), *Bmi-1* and *CCND1* (r = 0.6099, p<0.05) (Fig 4B), and *CDKN2B* and *CDKN2A* (r = 0.5585, p<0.05) (Fig 4C), and there was a strong correlation between the relative expression levels of *CDKN2B* and *CCND1* (r = -0.7967, p<0.01) (Fig 4D) (Table 3).

**Table 3:** Association of gene expression levels evaluated using pairwise combinations in grade III astrocytomas.

	*Bmi-1*	*CDC6*	*CDKN2B*	*CDKN2A*	*RB1*	*CCND1*
***Bmi-1***						
***CDC6***	0.1322					
***CDKN2B***	-0.5824*	-0.4319				
***CDKN2A***	-0.1568	-0.3802	0.5585*			
***RB1***	0.2967	0.1183	-0.3022	0.002751		
***CCND1***	0.6099*	0.03301	-0.7967**	-0.3466	0.3297	

Spearman's rho. (*p≤0.05, **p <0.01; ***p <0.001).

**Fig 5:**
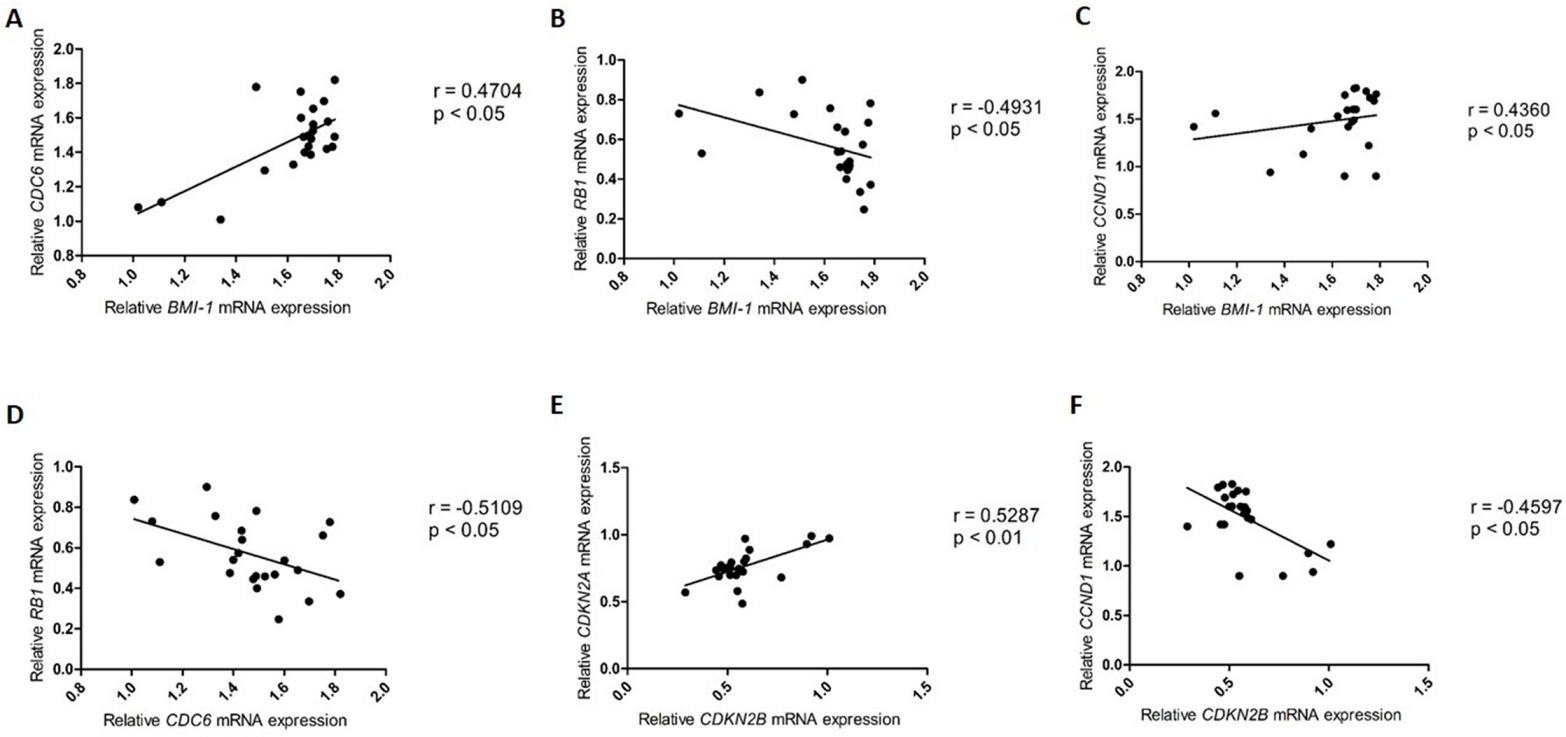
Correlation of the relative expression levels in grade IV astrocytomas. **(A)** *Bmi-1* and *CDC6*; **(B)** *Bmi-1* and *RB1* **; (C)** *Bmi-1* and *CCND1* **; (D)** *CDC6* and *RB1* **; (E)** *CDKN2B* and *CDKN2A* **; (F)** *CDKN2B* and *CCND1*; and **(G)** *CCND1* and *RB1*.

However, the analysis of grade IV astrocytomas indicated a moderate correlation between the relative expression levels of *Bmi-1* and *CDC6* (r = 0.4704, p<0.05) (Fig 5A), *Bmi-1* and *RB1* (r = -0.4931, p<0.05) (Fig 5B), *Bmi-1* and *CCND1* (r = 0.4360, p<0.05) (Fig 5C), *CDC6* and *RB1* (r = -0.5109, p<0.05) (Fig 5D), *CDKN2B* and *CDKN2A* (r = 0.5287, p<0.01) (Fig 5E), and *CDKN2B* and *CCND1* (r = -0.4597, p<0.01) (Fig 5F); there was a strong correlation between the relative expression levels of *CCND1* and *RB1* (r = -0.7667, p<0.001) (Fig 5G) (Table 4).

**Table 4:** Association of gene expression levels evaluated using pairwise combinations in grade IV astrocytomas.

	*Bmi-1*	*CDC6*	*CDKN2B*	*CDKN2A*	*RB1*	*CCND1*
***Bmi-1***						
***CDC6***	0.4704*					
***CDKN2B***	-0.2263	0.003953				
***CDKN2A***	-0.1067	-0.1245	0.5287**			
***RB1***	-0.4931*	-0.5109*	0.2085	-0.1215		
***CCND1***	0.4360*	0.3613	-0.4597*	-0.005932	0.7667***	-

Spearman's rho. (*p≤0.05, ** p<0.01; *** p<0.001).

### Absence of methylation of the promoter regions of *CDKN2B*, *CDKN2A*, *CDC6*, *Bmi-1*, and *RB1*

The sequencing results indicate that all the fragments of the promoter regions evaluated were not methylated and that, consequently, there was no association of this state with histological grade, age, or gender or with the relative expression of the transcripts analyzed.

## Discussion

In the present study, the relative expression of the genes *CDKN2B*, *CDKN2A*, *CDC6*, *Bmi-1*, *CCND1*, and *RB1* was evaluated at the transcript level, and these results were correlated with their methylation pattern. These genes are directly involved in the RB/E2F pathway, which functionally coordinates diverse biological processes, including cell differentiation, migration, growth, apoptosis, DNA replication, mitosis, DNA repair, and cell cycle checkpoints [10, 11, 12].

Previous studies have shown that genes associated with the RB1/E2F pathway are commonly deregulated in many human cancers via genetic or epigenetic mechanisms, resulting in the activation of E2F and leading to the transcription of several genes that regulate cell growth [29, 42–44].

### Methylation and expression of the genes *CDKN2A*, *CDKN2B*, and *RB1* in astrocytomas

The genes *CDKN2A*, *CDKN2B*, and *RB1* function as tumor suppressors; their decreased expression occurs via several mechanisms, including methylation, and contributes to the development of neoplasias [29, 45–52].

In our study, the genes *CDKN2B*, *CDKN2A*, and *RB1* were not methylated and hipoexpressed in all the tumor grades analyzed.

The results in the literature differ in relation to the pattern of methylation of the *CDKN2A* promoter region in astrocytomas [53–55]. Our results are consistent with those of Muñoz et al. [53], who evaluated hypermethylation frequency in the promoter region of five tumor suppressor genes, including *CDKN2A*, in astrocytomas and in cell culture lines. These authors found no evidence of hypermethylation in *CDKN2A*, suggesting that hypermethylation may not be an important mechanism in the activation of this gene in astrocytomas, considering that *CDKN2A* is primarily inactivated in astrocytomas via mutations and homozygous deletions [53].

In contrast, the absence of methylation of *CDKN2B* found in our study is consistent with the results of Yu et al. [56], who used methylation-specific PCR (MSP) in conjunction with the sequencing of 17 genes, including *CDKN2B*, in astrocytoma samples. These authors found that *CDKN2B* exhibited a uniform non-methylated pattern in all astrocytoma grades. In addition, our results corroborate those of Ohta et al. [57], who used the MSP and immunohistochemistry techniques in malignant astrocytic tumors (grades III and IV). These authors found that the promoter region of *CDKN2B* is hypermethylated in a very small percentage of cases (7%) and that methylation in *CDKN2B* was not associated with patient survival or with any clinicopathological characteristic, including tumor grade, cell proliferation activity, and responsiveness to adjuvant therapy. Our results are also consistent with those of studies that suggest that *CDKN2B* is commonly inactivated by homozygous deletions [58, 59] and, less frequently, via methylation of the promoter region [6].

Despite the findings on the methylation pattern, the real-time PCR results indicated that *CDKN2A* and *CDKN2B* were 1.3-fold underexpressed in GBM (Fig 3B). These findings are corroborated by those of Zolota et al. [60], who showed via immunohistochemistry that *CDKN2A* is most commonly expressed in low-grade astrocytic tumors compared with high-grade tumors.

The underexpression in high-grade astrocytomas often occurs because of the loss of chromosome 9p [59, 61, 62]. In addition, the underexpression of *CDKN2A* in high-grade tumors may be associated with other factors, including cellular control via microRNAs and cell cycle regulatory proteins, such as those of the Polycomb group [63]. With regard to *CDKN2B*, a few studies have investigated its expression in astrocytic tumors, indicating that the known mechanisms that can change its expression are loss of heterozygosity (LOH), homozygous deletion, point mutations, and hypermethylation of the promoter region [64].

The *RB1* gene is crucial for cellular control mechanisms, including cell proliferation and differentiation, apoptosis, and senescence [65], and several human tumors exhibit mutations, homozygous deletions, and methylation in the *RB1* promoter [66–68]. The functional loss of this gene disrupts checkpoints in G1, changes the rate of autophagy, apoptosis, angiogenesis, and metastasis, and causes structural defects in the centromeric region of chromosomes, affecting the recruitment of chromatin components (cohesin and condensin II) [68] and increasing genomic instability, chromosome and subchromosome ploidy (local amplifications and gains and losses of chromosomal arms), and error rates in chromosome segregation [68–70].

In astrocytomas, loss of heterozygosity in *RB1* is the most common event and is associated with increased tumor cell proliferation and decreased survival in more than one third of patients [71].

Although hypermethylation of the *RB1* promoter region is the second most common event in astrocytomas, our results indicate that *RB1* was not methylated in all the samples analyzed, regardless of the tumor grade. Bello et al. [72] demonstrated that the methylation frequency is 19% in pilocytic astrocytomas, 35% in grade II astrocytomas, and 13% in anaplastic astrocytomas. In GBM, the frequency of hypermethylation varies between 15% and 43% [73]. The discrepancy between our methylation results and those of other studies may have occurred because we analyzed a different fragment of the promoter region or because of the different techniques used; the two aforementioned studies used the MSP technique, whereas we used the BSP technique, which is considered more accurate than MSP.

### Methylation and expression of *CCND1*, *CDC6*, and *Bmi-1* in astrocytomas

The genes *CCND1*, *CDC6* and *Bmi-1*, when overexpressed, promote the proliferation of tumor cells. In our study, all these genes were not methylated and overexpressed in all the tumor grades analyzed.

The human *CDC6* gene codes for an AAA^+^ ATPase that binds to the replication origin recognition complex (ORC) and facilitates the recruitment of the mini-chromosome maintenance (MCM) complex [25]. After the formation of this complex and the subsequent binding of regulatory factors and replication fork components, double-stranded DNA opens for replication. The phosphorylation of the N-terminal domain of CDC6 exposes the nuclear export sequence (NES), transporting it to the cytoplasm during the G2 and M phases. During mitosis, the CDC6 protein is degraded by the ubiquitin-proteasome system.

Our results indicate that the overexpression of *CDC6* is associated with increased tumor grade. The overexpression of *CDC6* induces re-replication [74, 75], which is a form of replication stress, results in increased genomic instability and promotes malignant behavior [74, 76–79]. High levels of CDC6 protein were reported in 55% of brain tumors [80]. *CDC6* is also overexpressed in 50% of cases of non-small cell lung cancer [76] and mantle cell lymphoma [81].

On the basis of these results and on the premise that human *CDC6* is essential for initiation of DNA replication [82], we can infer that the overexpression of *CDC6* in astrocytic tumors can aid in cell cycle progression and progression from low-grade to high-grade tumors. Cell culture studies have shown that the suppression of *CDC6* in G1 prevents cells from entering into the S phase [83, 84]. Furthermore, recent studies have found that *CDC6* silencing by RNA interference (RNAi) prevents cell proliferation and induces apoptosis [85, 86].

To the best of our knowledge, this is the first study that evaluated the methylation pattern of *CDC6* in astrocytomas. However, our results are contrary to those reported by Bastian et al. [87], who reported the occurrence of hypermethylation of the *CDC6* promoter in prostate tumors. In addition, Jin and Fondel [88] suggest that a major mechanism of regulation of *CDC6* expression involves changes in the patterns of histone methylation and acetylation.

The *Bmi-1* gene is a member of the Polycomb 1 (PcG1) gene cluster and functions as a transcriptional repressor of several genes via acetylation, methylation, and mono-ubiquitination of histones and methylation of chromatin [89].

Increasing evidence indicates that *Bmi-1* is overexpressed in several cancer types, including leukemia, hepatocellular carcinoma, laryngeal carcinoma, lung cancer, breast cancer, and colon cancer [90–97].

Our gene expression results corroborate these findings, showing that *Bmi-1* was overexpressed in all tumor grades evaluated; this expression was 1.15-fold higher in GBM compared with that in grade II astrocytomas. Therefore, the increase in tumor grade is positively correlated with the prognosis of patients with increased expression of this gene.

Li et al. [98] demonstrated that *Bmi-1* was overexpressed in 93.9% of glioma samples from 297 patients evaluated and that this expression was inversely correlated with the survival time of patients and positively correlated with disease prognosis. In addition, the authors demonstrated that *Bmi-1* confers resistance to apoptosis in glioma cells via the IKK-NF-kB pathway, suggesting that this is a useful prognostic marker for gliomas.

Mihic-Probs et al. [99] reported that in 64% of primary melanomas and in 71% of metastatic melanomas, *Bmi-1* is overexpressed and associated with the clinical course of the disease. Recent studies have found a positive correlation between *Bmi-1* levels and survival and recurrence in patients with tongue cancer, squamous cell carcinoma of the oropharynx, and non-small cell lung cancer [100, 101].

It is known that *Bmi-1* promotes cell proliferation by suppressing the RB pathway [102]. We also found a negative correlation between the relative expression levels of *CDKN2B* and *Bmi-1*. Although a direct association between these two genes has not been reported, it is known that in human squamous cell carcinoma of the head and neck, there is a negative correlation between high levels of *Bmi-1* and the regulatory transcriptional region in the INK4-ARF locus, known as the regulatory domain (RD), resulting in changes in the transcriptional levels of *CDKN2A* and *CDKN2B* [103].

The *CCND1* gene is part of the cyclin family; cyclins bind to and activate CDKs, phosphorylate pRB, and, ultimately, induce the transcription of several genes necessary for cell entry into the S-phase [104].

D-type cyclins are highly important in the assessment of several cancer types because they have a positive effect on several oncogenic pathways [105, 106]. In tumors, many studies have reported that this gene may undergo amplification, rearrangements, and overexpression or undergo methylation in the promoter region [6, 64, 106, 107].

Our real-time PCR results indicate that *CCND1* was overexpressed in 58.3% of cases (35/60) and that when the relative expression of this transcript was correlated with histological astrocytoma grades, its expression levels were approximately 5-fold higher in grades III and IV tumors compared with the levels in grade II tumors. The overexpression of *CCND1* is one of the most frequently observed changes in various cancer types [108]. Moreover, it is known that overexpression of *CCND1* results in RB dysfunction, resulting in the promotion of cell proliferation, which is considered a hallmark of carcinogenesis [109, 110].

Wang et al. [111] infected two glioblastoma cell lines (SHP-44 and U251) with shRNA and observed that silencing of *CCND1* inhibited cell proliferation, induced apoptosis, and increased their invasive capacity. In contrast, the overexpression of *CCND1* increased the proliferation and invasive capacity of both human glioblastoma cell lines but reduced apoptosis. Therefore, the ability to suppress the malignant phenotype by decreasing *CCND1* expression may provide a new approach for gene therapy in patients with glioblastoma.

The functional interactions of the members of RB/E2F pathway have been elucidated. In G0 and in the early stages of G1, RB is hypophosphorylated and forms complexes with members of the E2F family of transcription factors. The complexes formed prevent E2F from binding to the promoters of the genes associated with the G1/S transition [67]. Once engaged in cell proliferation, pRB is progressively hyperphosphorylated by CDK4 and CDK6 in the late G1 phase, promoting progression to the S phase. The INK4 family proteins (p16^INK4A^ and p15^INK4B^) bind to and inhibit the activity of CDK4 and CDK6, maintaining pRB in a hypophosphorylated state [112]. In addition, p16^INK4A^ and p15^INK4B^ compete with D-type cyclins for CDK4/6 to prevent the formation of active kinase complexes. In this pathway, proteins of the INK4 and RB families function as tumor suppressors, whereas D-type cyclins, CDK4/6, and E2F promote tumor proliferation [43].

Our results suggest that there is an intense, coordinated deregulation of the entire RB/E2F pathway that is associated with tumor progression and patient prognosis. Despite the need for further studies with a larger sample size to confirm our results, we suggest that evaluation of the gene expression levels of members of this pathway can be used in the monitoring of patients with astrocytomas in clinical practice and for the prognostic indication of disease progression.
